# Fixational eye movements abnormalities and rate of visual acuity and stereoacuity improvement with part time patching

**DOI:** 10.1038/s41598-020-79077-5

**Published:** 2021-01-13

**Authors:** Matteo Scaramuzzi, Jordan Murray, Paolo Nucci, Aasef G. Shaikh, Fatema F. Ghasia

**Affiliations:** 1 Cole Eye Institute, Cleveland Clinic, Cleveland, OH USA; 2 Department of Neuroscience, Unit of Ophthalmology, IRCCS Istituto Giannina Gaslini, Genoa, Italy; 3 DISCCO, University of Milan, Milan, Italy; 4 Daroff—Dell’Osso Ocular Motility Laboratory, Cleveland, OH USA; 5 Case Medical Center, Case Western Reserve University, Cleveland, OH USA

**Keywords:** Medical research, Eye diseases, Ocular motility disorders

## Abstract

Residual amblyopia is seen in 40% of amblyopic patients treated with part-time patching. Amblyopic patients with infantile onset strabismus or anisometropia can develop fusion maldevelopment nystagmus syndrome (FMNS). The purpose of this study was to understand the effects of presence of FMNS and clinical subtype of amblyopia on visual acuity and stereo-acuity improvement in children treated with part-time patching. Forty amblyopic children who had fixation eye movement recordings and at least 12 months of follow-up after initiating part-time patching were included. We classified amblyopic subjects per the fixational eye movements characteristics into those without any nystagmus, those with FMNS and patients with nystagmus without any structural anomalies that do not meet the criteria of FMNS or idiopathic infantile nystagmus. We also classified the patients per the clinical type of amblyopia. Patching was continued until amblyopia was resolved or no visual acuity improvement was noted at two consecutive visits. Children with anisometropic amblyopia and without FMNS have a faster improvement and plateaued sooner. Regression was only seen in patients with strabismic/mixed amblyopia particularly those with FMNS. Patients with FMNS had improvement in visual acuity but poor stereopsis with part-time patching and required longer duration of treatment.

## Introduction

Amblyopia arises due to the disruption in the correlated activity of the two eyes during the critical periods of vision development^1,2^. Neurophysiologic studies suggest that the effects of the de-correlated binocular signals on the visual cortex are most significant if they occur at the emergence of stereoacuity in early infancy^3–5^. Non-human primate model studies have revealed that the loss of horizontal binocular connections within area V1 in infancy results in the development of latent nystagmus^6–10^, now referred to as Fusion Maldevelopment Nystagmus Syndrome (FMNS)^11^. FMNS is commonly associated with infantile strabismus, but monocular deprivation and high anisometropia that causes binocular de-correlation in early infancy can produce FMNS^7,12^.

Patching therapy is commonly employed, but up to 40% of treated children have residual amblyopia, and 25% have regression^13,14^. Some risk factors associated with residual/recurrent amblyopia include severe amblyopia and older age at the time of diagnosis, strabismic amblyopia, lower (younger) age at the end of treatment, and abrupt cessation, particularly of full-time patching^15–19^. Other possible causes that could be associated with treatment response include the presence of FMNS and increased fixation instability seen in amblyopia patients^20–26^. The patching therapy was considered to be contraindicated in patients with FMNS because the intensity (amplitude × frequency) of FMNS increases under monocular viewing conditions^27,28^. A successive study in a small cohort of patients showed that a significant improvement in visual acuity could be obtained in patients with FMNS with full-time patching during all waking hours^29^. Simonsz et al. recorded eye movements in five patients with FMNS before and after 2 days of full-time patching of the fellow eye. They found a reduction in slow phase velocity when the amblyopic eye was fixing post occlusion^30^. Thus, these studies provide evidence that full-time patching therapy can improve visual acuity in FMNS patients. Birch et al. have described an association between abnormal stereo-acuity and patients with FMNS. They found that 67% of all children with abnormal stereo-acuity had FMNS waveform, and nearly all children with nil stereo-acuity had FMNS waveforms^26^. Bosworth and Birch^31^ have found that the risk for persistent amblyopia was greater among children with nil stereo-acuity than those with measurable stereoacuity at treatment onset. The current standard of amblyopia treatment comprises of part-time patching ranging from 2 to 6 h/day per the amblyopia severity as per the guidelines from the landmark PEDIG Amblyopia Treatment Studies^32–34^. Little is known to date about the association between stereo-acuity and part-time patching treatment in FMNS.

In the current manuscript, we measured fixation eye movements at the end of part-time patching treatment and analyzed the rate of improvement of visual acuity and stereoacuity improvement in amblyopia patients with and without FMNS. We hypothesize that the presence of FMNS, would be associated with a slower rate of visual acuity improvement in amblyopic patients treated with patching therapy which requires monocular viewing compared to patients without FMNS. We also hypothesize that the presence of FMNS will be associated with minimal/no change in stereoacuity, as FMNS is a hallmark of binocular maldevelopment in early infancy. We also analyzed the regression of amblyopia in patients after stopping part-time patching.

## Results

### Clinical type of amblyopia

The study subjects were categorized based on the clinical type (anisometropia: n = 15, strabismic n = 3, mixed n = 22). The age of the start of patching treatment was similar in patients with mixed/strabismic amblyopia compared to anisometropic amblyopia (anisometropic: 75 ± 17 months vs mixed/strabismic: 64 ± 29 months, p = 0.36). Similarly, there was no difference in visual acuity at the time of diagnosis per the clinical types of amblyopia (anisometropic = 0.64 ± 0.42 logMAR, strabismic/mixed = 0.53 ± 0.26 logMAR, p = 0.54), while there was an expected significant difference in stereoacuity (anisometropic = 2.09 ± 0.66 logarcsec vs. strabismic/mixed = 2.82 ± 0.87 logarcsec, p = 0.002). Eight patients required strabismus surgery (Table 1). There was no difference in age (surgery vs no surgery: 54 ± 33 months vs 66 ± 21 months, Mann Whitney U test p = 0.47), visual acuity (surgery vs no surgery: 0.60 ± 0.30 log MARvs 0.58 ± 0.34 logMAR, Mann Whitney U Test p = 0.48) and stereoacuity (surgery vs no surgery: 3.05 ± 0.85 logarcsec vs 2.45 ± 0.85 logarcsec, p = 0.693) at the time of diagnosis between patients with strabismus requiring surgery versus those that did not require surgery.

**Table 1:** Demographics, ophthalmic exam and strabismus surgery data of the enrolled subjects.

ID	Gender	Age at patching (duration) (months)	Category at time of patching	Visual Acuity at time of patching (LogMAR)	Stereoacuity at time of patching (arc second)	Eye movement characteristics	Refraction (RE; LE) (diopters)	Strabismus (near; distance) (prism diopters)	Surgery and age (years)
1	F	27 (30)	Strabismic	1.4	Nil	None	+ 6.5	ET 30	BMR
			Severe	0.3			+ 6.25	ET 30	REC
									Age 3
2	M	69 (26)	Mixed	0.5	200	None	+ 5	ET 35	R&R RE
			Moderate	0			+ 1	ET 35	Age 6
3	F	82 (18)	Mixed	0.4	100	None	+ 3.0 + 1.25 × 65	ET 12	
			Moderate	0.1			+ 1.25 + 0.25 × 115	E(T) 4–6	
4	M	44 (48)	Mixed	0.2	140	None	+ 2.5	Ortho with glasses	
			Moderate	0.5			+ 4.5		
5	M	84 (9)	Mixed	0	100	None	Plano + 0.50 × 95	XT 20	
			Moderate	0.4			− 0.75 + 3.5 × 85	XT 30	
6	M	46 (75)	Mixed	0	140	None	+ 6.5 + 2.00 × 70	Ortho with glasses	
			Severe	0.8			+ 0.5 + 0.5 × 90		
7	F	41 (22)	Mixed	0.1	50	None	+ 5.5 + 1.00 × 100	Ortho with glasses	
			Moderate	0.3			+ 6.5 + 1.0 × 80		
8	F	77 (21)	Mixed	0	Nil	None	+ 4.50 + 2.00 × 90	E(T) 8	
			Moderate	0.3			+ 5.5 + 2.25 × 90	E(T) 10	
9	M	79 (6)	Anisometropic	0	Nil	None	Plano + 0.75 × 95	Ortho	
			Moderate	0.5			+ 4.25 + 2.00 × 90		
10	F	66 (14)	Anisometropic	0.5	80	None	+ 5 + 0.50 × 100	Ortho	
			Moderate	0.1			+ 3 + 0.50 × 80		
11	F	60 (19)	Anisometropic	0.7	50	None	+ 7.5	Ortho	
			Severe	0.1			+ 5.0 + 0.50 × 180		
12	M	53 (15)	Anisometropic	0.55	40	None	+ 4 + 0.50 × 105	Ortho	
			Moderate	0.2			+ 0.5 + 0.5 × 85		
13	F	117 (6)	Anisometropic	0	40	None	− 0.25 + 0.5 × 90	Ortho	
			Mild	0.2			Plano + 2 × 85		
14	F	81 (6)	Anisometropic	0.4	60	None	− 2.75 + 4.25 × 95	Ortho	
			Moderate	0			+ 1.5		
15	F	53 (21)	Anisometropic	0.1	60	None	+ 0.5 + 1.00 × 90	Ortho	
			Moderate	0.6			+ 3.5 + 1.00 × 90		
16	F	63 (6)	Anisometropic	0.4	40	None	+ 4.25 + 1.0 × 95	Ortho	
			Moderate	0			+ 1.75 + 0.25 × 80		
17	M	90 (30)	Anisometropic	0.2	50	None	Plano + 0.50 × 85	Ortho	
			Moderate	0.5			+ 5.25 + 2.00 × 105		
18	M	90 (22)	Anisometropic	1.2	140	None	+ 7.00 + 0.50 × 60	Ortho	
			Severe	0			+ 1.00 + 0.25 × 50		
19	M	39 (21)	Strabismic	0	140	Nystagmus No	+ 2.75 + 0.50 × 180	ET 35	BMR
			Moderate	0.3		FMNS	+ 2.75 + 0.50 × 180	ET 35	REC
									Age 3
20	F	83 (17)	Mixed	0.2	100	Nystagmus No	− 1.75 + 3 × 85	Ortho with glasses	
			Moderate	0.6		FMNS	− 10.00 + 3.75 × 85		
21	F	66 (11)	Mixed	0.3	Nil	Nystagmus No	+ 2.25 + 0.75 × 80	Ortho with glasses	
			Severe	0.7		FMNS	+ 3.5 + 0.5 × 135		
22	M	63 (27)	Mixed	0.2	Nil	Nystagmus No	+ 1.25 + 0.75 × 110	Ortho with glasses	
			Mild	0		FMNS	+ 0.25 + 2.0 × 80		
23	F	95 (28)	Mixed	0.4	200	Nystagmus No	− 11.5 + 0.75 × 75	XT 14	RLR
			Moderate	0.1		FMNS	− 6.5 + 1.0 × 105	XT 25	REC
									Age 8
24	M	102 (28)	Mixed	0.1	100	Nystagmus No	+ 6 + 2.0 × 90	Ortho with glasses	
			Mild	0.2		FMNS	+ 7 + 1.75 × 90		
25	M	33 (31)	Mixed	0.2	Nil	Nystagmus No	+ 1.50	LE(T) 8	
			Severe	0.7		FMNS	+ 4	LE(T) 10	
26	F	84 (20)	Mixed	0.1	200	Nystagmus No	− 0.75 + 0.5 × 75	XT 20	RLR
			Moderate	0.4		FMNS	+ 1.5 + 1.00 × 90	XT 25	REC
									Age 8
27	M	85 (6)	Mixed	0	80	Nystagmus no	+ 1.25 + 1.5 × 100	Flick X(T)	
			Moderate	0.6		FMNS	+ 4.00 + 2.00 × 70	6 LX(T)	
28	M	63 (34)	Anisometropic	0.1	140	Nystagmus No	+ 0.25 + 0.5 × 90	Ortho	
			Severe	1.9		FMNS	− 10.75 + 2.0 × 50		
29	M	80 (39)	Anisometropic	0.2	100	Nystagmus No	+ 7.25 + 1.5 × 90	Ortho	
			Moderate	0.4		FMNS	+ 8.25 + 1.5 × 100		
30	M	75 (31)	Anisometropic	0.8	200	Nystagmus No	+ 6.75 + 3 × 90	Ortho	
			Severe	0		FMNS	+ 0.5		
31	F	71 (16)	Anisometropic	0.4	100	Nystagmus No	+ 4 + 1.25 × 85	Ortho	
			Moderate	0		FMNS	+ 1.5 + 0.5 × 85		
32	F	81 (25)	Anisometropic	0	80	Nystagmus No	+ 1.00 + 0.5 × 90	Ortho	
			Moderate	0.5		FMNS	+ 3.75		
33	F	14 (45)	Strabismic	0.7	Nil	FMNS	+ 3.50 + 1.75 × 90	ET 45	BMR
			Severe	0.2			+ 3.50 + 1.75 × 90	ET 45	REC
									Age 1
34	M	72 (20)	Mixed	0.2	Nil	FMNS	+ 5.00 + 0.50 × 90	Ortho with glasses	
			Severe	0.7			+ 6.25 + 1.00 × 95		
35	M	80 (6)	Mixed	0.55	Nil	FMNS	− 9.5 + 2.5 × 165	ET 4	
			Moderate	0			plano + 0.75 × 45	ET 4	
36	F	67 (38)	Mixed	0	Nil	FMNS	+ 5 + 1.5 × 80	Ortho with glasses	
			Moderate	0.6			+ 6 + 1.5 × 95		
37	M	83 (25)	Mixed	0.4	Nil	FMNS	− 6.75 + 3.75 × 90	XT 25	BLR
			Severe	0.8			− 9.0 + 3.75 × 90	XT 45	REC
									Age 8
38	M	18 (54)	Mixed	0.3	Nil	FMNS	+ 4.5	E(T) 30	BMR
			Severe	0.2			+ 3.5	ET 25	REC
									Age 3
39	F	60 (38)	Mixed	0.3	400	FMNS	+ 4	XT 20	
			Moderate	0.1			+ 2.25	XT 20	
40	M	30 (79)	Mixed	0.5	200	FMNS	+ 8.00 + 1.5 × 90	ET 6–8	
			Moderate	0.3			+ 7.25 + 0.5 × 90	ET 4	

*BLR* bilateral lateral recti muscles, *BMR* bilateral medial recti muscles, *CC* with correction, *ET* esotropia, *E(T) *intermittent esotropia, *F* female, *FMNS* fusion maldevelopment nystagmus syndrome, *LE* left eye, *M* male, *RE* right eye, *REC* recession, *R&R* recession and resection, *RLR* right lateral rectus recession, *XT* exotropia, *X(T) *intermittent exotropia.

### Eye movement characteristics

**Figure 1:**
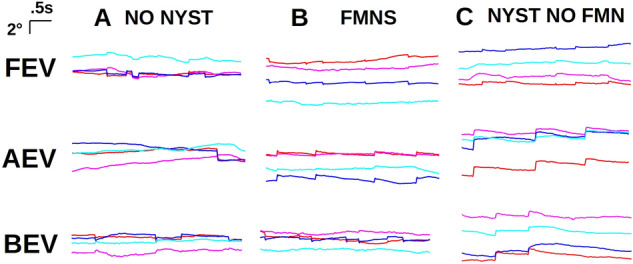
Eye movement records. Eye positions vs time for patients without nystagmus (**A**), FMNS (**B**), and with nystagmus but not FMNS (**C**), during fellow eye viewing (top row), amblyopic eye viewing (middle row), and both eyes viewing (bottom row). Waveforms are plotted with a common scale indicated at upper left. Red: right horizontal, blue: left horizontal, magenta: right vertical, cyan: left vertical. The positive vertical axis corresponds to rightward and upward eye movements. Note the reversal in direction between FEV and AEV in FMNS patients that is not present in nystagmus without FMN patient. Note also the greater intensity during AEV and the absence of acceleration during slow phases in nystagmus patients both with and without FMNS.

The fixational eye movement traces obtained at the end of treatment were evaluated, and amblyopic patients were classified based on the presence or absence of nystagmus (Fig. 1)^35^. Patients without nystagmus (no nystagmus—Fig. 1A) exhibited alternating fixational saccades with inter-saccadic drifts, similar to healthy subjects^23,36,37^. Patients with nystagmus were further classified into those with FMNS (Fig. 1B) versus those that did not meet the criteria of FMNS (Fig. 1C). The presence of FMNS was determined based on the classic reversal in the quick phase of nystagmus with linear/decreasing velocity nasally directed slow phase observed during monocular viewing conditions^28^. Patients with nystagmus/nystagmus like movements who did not exhibit the classic reversal in the direction of quick phases were characterized as Nystagmus without FMNS (Nyst no FMN). These patients had jerk nystagmus with dynamic overshoots of quick phases and differed from Infantile Nystagmus Syndrome patients in that their velocity was decreasing or linear, unlike the increasing eye velocity characteristics seen in patients with Infantile Nystagmus Syndrome. Also, patients with nystagmus but no FMNS did not have the Dissociated Vertical Deviation frequently seen in FMNS patients. The fixational eye movements were evaluated, and amblyopic patients were classified into those with no nystagmus (n = 18), those with FMNS (n = 8), and those with nystagmus but without the classic reversal in the quick phase of nystagmus seen in FMNS (n = 14). There was no difference in the age of the start of patching per the fixational eye movement characteristics (No nystagmus = 68 ± 22 months, Nystagmus no FMNS = 73 ± 19 months, FMNS = 60 ± 41 months, p = 0.38). Per the fixation eye movement characteristics, there was no difference in visual acuity at the time of diagnosis (None = 0.52 ± 0.23 logMAR, Nystagmus no FMNS = 0.62 ± 0.43 logMAR and FMNS = 0.50 ± 0.16 logMAR, p = 0.9), while there was a significant difference in stereoacuity (None = 2.05 ± 0.56 logarcsec, Nystagmus no FMNS = 2.56 ± 0.84 logarcsec and FMNS = 3.49 ± 0.65 logarcsec, p = 0.001).

### Improvement and type of amblyopia

**Figure 2:**
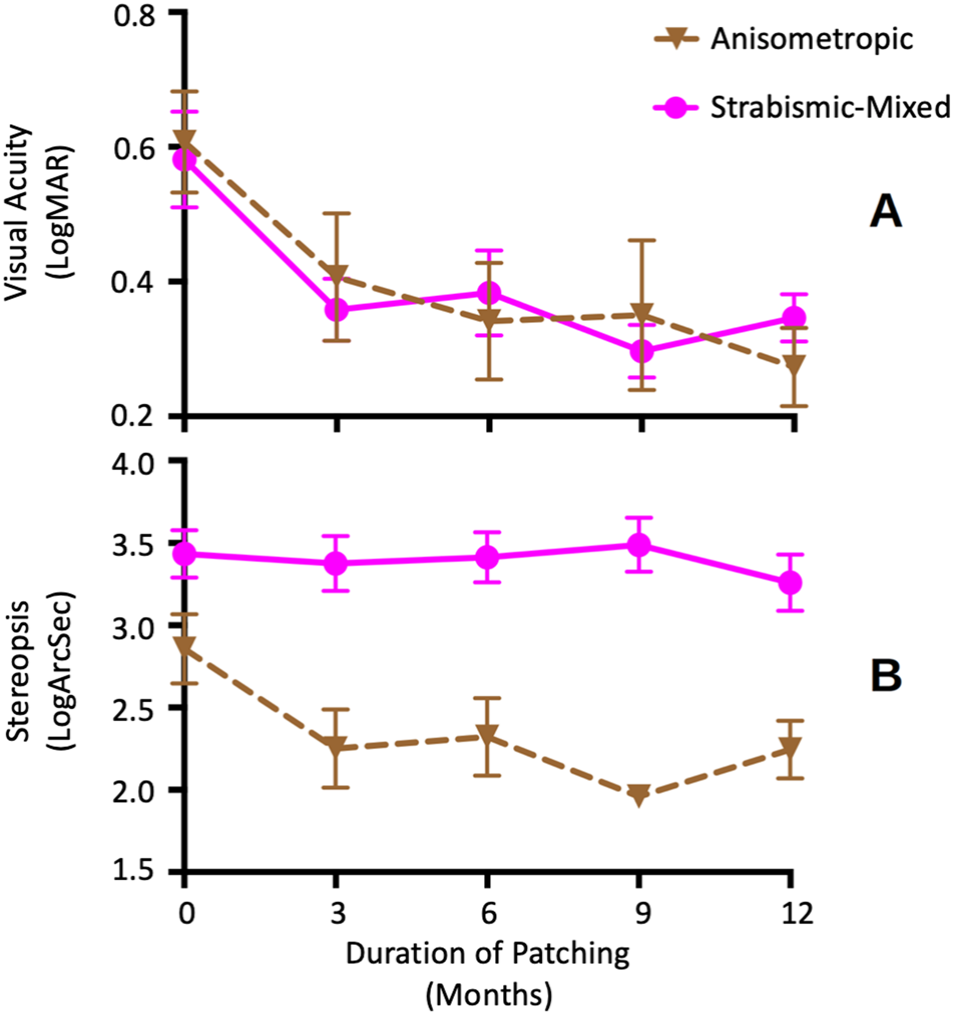
Visual acuity and stereoacuity per type of amblyopia. The mean and standard error of mean of visual acuity (**A**) and stereoacuity (**B**) change sub-grouped by the type of amblyopia at baseline and during the first year after initiating patching treatment. The lower scores indicate better visual acuity (log MAR) and stereoacuity (log arc sec). visual acuity and stereoacuity improvement was greater in anisometropic than strabismic/mixed group. Brown triangles—dashed line: anisometropic; magenta circles—full line: strabismic/mixed.

Figure 2A,B plot the average and stdev of visual acuity and stereo-acuity in the anisometropic and mixed/strabismic groups at baseline and within the first year after initiating patching treatment. To determine statistical significance per different groups, we employed mixed-effect model with random intercepts. We found that the visual acuity change within the first year of patching treatment was found to be significantly different between the anisometropic versus strabismic/mixed amblyopia type (mixed effect model: F = 5.9, p = 0.016). Both groups had improvement in visual acuity (indicated by negative values). The average beta coefficient of visual acuity change ((log MAR visual acuity/3 months of patching treatment) was greater in patients with anisometropia (− 0.025 ± 0.01) compared to the strabismic/mixed amblyopia group (− 0.012 ± 0.02). We also found that the change of stereo-acuity within the first year of patching treatment was significantly different between anisometropic versus strabismic/mixed groups (mixed effect model: F = 8.8, p = 0.005). The average beta coefficient of stereo-acuity change (log arcsec/3 months of patching treatment) was greater in the anisometropic group (− 0.05 ± 0.08) compared to strabismic/mixed amblyopia type (0.003 ± 0.04). A positive value indicates a lack of improvement in the strabismic/mixed group.

### Improvement and fixational eye movements characteristics

**Figure 3:**
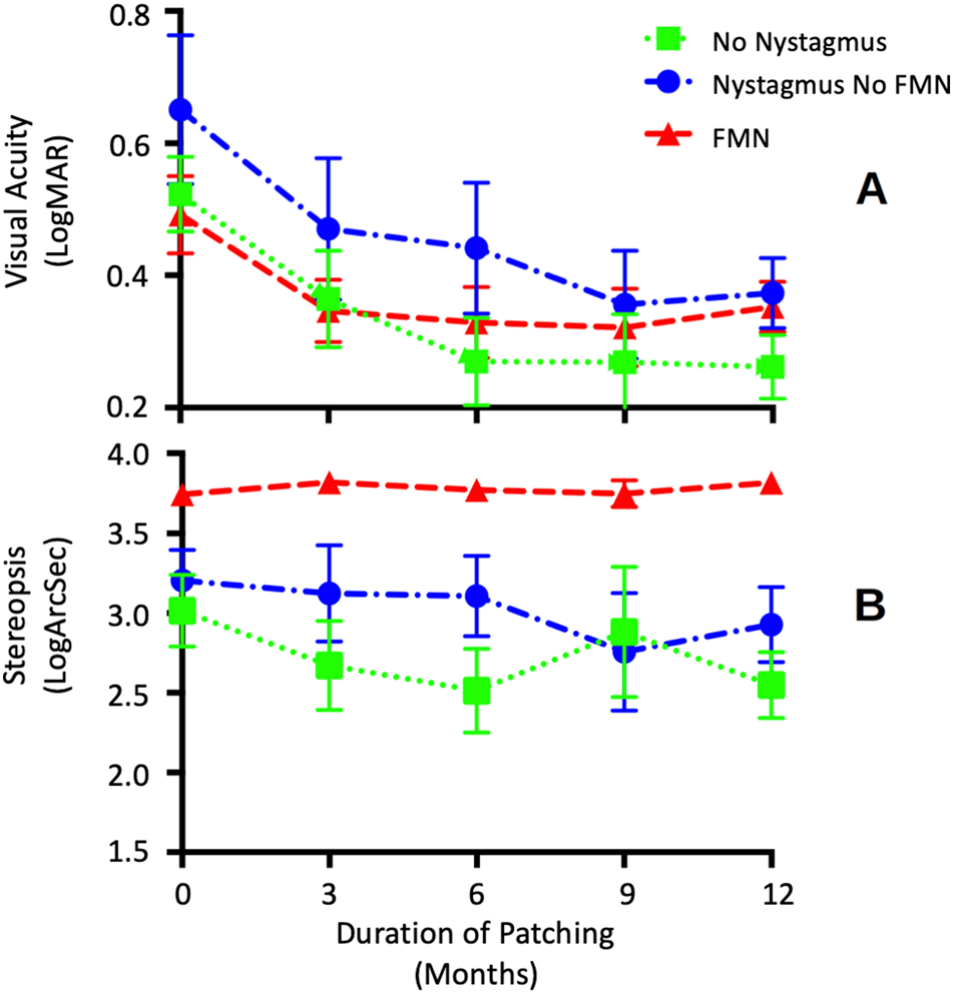
Visual acuity and stereoacuity per fixational eye movements characteristcs. The mean and standard error of mean of visual acuity (**A**) and stereoacuity (**B**) change sub-grouped by the fixation eye movement characteristics at baseline and during the first year after initiating patching treatment. The lower scores indicate better visual acuity (log MAR) and stereoacuity (log arc sec). Visual acuity and stereoacuity improvement was greater in patients without nystagmus. No improvement of stereoacuity was recorded in FMNS group. Green squares: no nystagmus; blue circles: nystagmus no FMNS; red triangles: FMNS.

Figure 3A,B plots the average and stdev of visual acuity and stereo acuity for each subgroup at baseline and within the first year after initiating patching treatment. To determine statistical significance between different groups, we employed a mixed-effect model with random intercepts. We found that the change of visual acuity within the first year of patching treatment was found to be significantly different between no nystagmus, nystagmus without FMNS, and FMNS groups (mixed effect model: F = 4.3, p = 0.04). All three groups had improvement in visual acuity (indicated by negative values). The average beta coefficient of visual acuity change (log MAR visual acuity/3 months of patching treatment) was greater in patients with no nystagmus (− 0.025 ± 0.01) compared to nystagmus no FMNS (− 0.018 ± 0.02) and FMNS (− 0.01 ± 0.01) groups. We also found that the change of stereoacuity within the first year of patching treatment was found to be significantly different between no nystagmus, nystagmus without FMNS, and FMNS groups (mixed effect model: F = 5.8, p = 0.02). The average beta coefficient of stereoacuity change (log arcsec/3 months of patching treatment) was greater in no nystagmus group (− 0.049 ± 0.09) compared to nystagmus without FMNS (− 0.015 ± 0.03) group with no improvement in the FMNS group (0.02 ± 0.04). A positive value indicates a lack of improvement in the FMNS group.

### No improvement and regression

In our cohort, 17% of patients had regression with a decrease in visual acuity after stopping patching treatment. All of the patients who experienced regression had strabismic/mixed amblyopia and had received patching treatment for at least 6 months before the treatment was discontinued due to a plateau in visual acuity or the resolution of amblyopia (defined as inter-ocular visual acuity difference of < 2 lines). Regression was noted in 2/18 patients without nystagmus, 1/14 patient with nystagmus without FMNS, and 4/8 patients with FMNS. Of the two patients without nystagmus who experienced regression (Subject 3 and 4), both patients regained their visual acuity on restarting the patching treatment and had mild residual amblyopia with some stereoacuity at the end of treatment. The patients with nystagmus without FMNS (Subject 22) and FMNS (Subjects 33, 38, 39, and 40) experienced regression and regained the visual acuity after restarting treatment but did not have any stereoacuity at the end of treatment.

10% of our cohort had no improvement in visual acuity post patching treatment. These include one subject with no nystagmus (Subjects 2), two with nystagmus without FMNS (Subject 23 and 27), and one with FMNS (Subject 35). Patching was started after age 5 in all four subjects, and all of them had high anisometropia (2 aniso-myopia, 2 with aniso-hyperopia). The two aniso-hyperopic patients opted for atropine penalization treatment.

### Visual acuity improvement and treatment plateau

The improvement in visual acuity and stereoacuity was analyzed between groups per the fixation eye movement characteristics and as a function of the clinical type of amblyopia. Patients who did not have any improvement in visual acuity with patching treatment were excluded from this analysis. Similar visual acuity improvement levels were found in patients with and without FMNS (no nystagmus: 0.39 ± 0.28, nystagmus no FMNS: 0.33 ± 0.36, FMNS: 0.29 ± 0.16 LogMAR, F = 0.30, p = 0.74). A similar analysis was performed as a function of the clinical type of amblyopia. The results were comparable between anisometropic versus strabismic/mixed groups (visual acuity improvement: anisometropia: 0.41 ± 0.31 and strabismic/mixed: 0.29 ± 0.26 LogMAR, t-test p = 0.29). Children with no nystagmus plateaued sooner in terms of visual acuity improvement compared to the no nystagmus group (no nystagmus: 9.3 ± 6.5; nystagmus no FMNS: 14.2 ± 8.2; FMNS: 30.2 ± 23.2 months, one way ANOVA, F = 6.4, p = 0.005). Children with anisometropic amblyopia plateaued sooner in terms of visual acuity improvement than strabismic/mixed amblyopia groups (anisometropia: 9.1 ± 6.8 months, strabismic/mixed: 22.1 ± 17 months, unpaired t test p = 0.01).

### Stereoacuity improvement and treatment plateau

21/40 patients had improvement in stereoacuity. The majority of patients without nystagmus had improvement in stereoacuity (14/18) compared to nystagmus no FMNS (6/14) and FMNS (1/8). The extent of stereoacuity improvement was analyzed in patients with and without nystagmus—we excluded FMNS patients from this analysis as only 1 FMNS patient had improvement in stereoacuity after 29 months of treatment. Of the patients that had improvement in stereoacuity, there was a trend that no nystagmus had greater improvement (1.3 ± 0.76 log arcsec) compared to nystagmus no FMNS group (0.96 ± 0.57 log arcsec, p = 0.15, Mann–Whitney U test). There was no difference in the time to reach the best possible stereoacuity in patients without nystagmus (26 ± 19 months) versus those with nystagmus without FMNS (20.5 ± 9.5 months, p = 0.5, Mann Whitney U test).

Of the 21 patients with improvement in stereoacuity, 14 had anisometropic amblyopia, and 7 had strabismic/mixed amblyopia. The extent of stereoacuity improvement was similar irrespective of amblyopia type (anisometropia: 1.2 ± 0.82 versus mixed/strabismic: 1.4 ± 0.30 log arcsec, Mann–Whitney U test p = 0.5). Like visual acuity improvement, patients with anisometropic amblyopia (19.7 ± 12.5 months) reached the best possible stereoacuity sooner with treatment than patients with strabismic/mixed amblyopia (34 ± 19.5 months, Mann Whitney test p = 0.02).

## Discussion

In this retrospective study, we characterized fixational eye movements at the end of treatment in amblyopic patients treated with part-time patching therapy and evaluated the rate of improvement in visual acuity and stereoacuity. We found that the presence of FMNS was associated with a slower rate of visual acuity improvement and poor recovery of stereopsis. Amblyopic patients with nystagmus without the reversal in the direction of the quick phase, as seen in FMNS, had a similar rate of improvement in visual acuity but less improvement in stereoacuity compared to patients without nystagmus. The velocity waveform of nystagmus differs from that seen in patients with idiopathic infantile nystagmus syndrome. Nystagmus that is not FMNS or INS, has been previously reported in patients with monocular vision loss in early childhood^38,39^. Amblyopic patients have increased drifts. Thus, reduced visual acuity due to amblyopia and the increased drifts interrupted by corrective saccades could result in the development of nystagmus beats in the absence of FMNS/INS. We found that the rate of visual acuity and stereoacuity improvement was faster in anisometropic amblyopia and plateaued sooner with part-time patching than strabismic/mixed amblyopia patients. Similarly, patients without nystagmus (both anisometropic and strabismic, or mixed, amblyopia) plateaued sooner than those with nystagmus. The patients who experienced regression had strabismic/mixed amblyopia and had received part-time patching treatment for at least 6 months before the treatment was discontinued. The risk of regression was greater in FMNS patients and required longer durations of treatment than amblyopic patients without FMNS.

### Effect of patching on visual acuity

A few studies describe the visual acuity improvement as a function of patching duration. Stewart et al. found an average visual acuity improvement of 0.35 logMAR from a cumulative dose of patching for 150–250 h irrespective of the type of amblyopia and a flattening of the dose/response curve after 400 h of treatment^40^. ATS2A and ATS2B^32,33^ did not find any differences in the extent of visual acuity improvement at 17 weeks in groups per the clinical type of amblyopia- however, they found that patients with worse initial visual acuity and age at treatment with children < 5 years of age had greater visual acuity improvement. In a large retrospective study of 877 patients recruited per the PEDIG ATS2 A and B inclusion criteria^34^, the authors found similar levels of visual acuity improvement, as reported in PEDIG studies. However, the treatment duration was longer, probably due to the differences in motivation for compliance and follow-ups between patients included in trials versus in real-world clinical practice. We have previously described the results of visual acuity and stereoacuity measurements obtained at the end of part-time patching therapy per the clinical type of amblyopia and per the presence of FMNS^41^. We found that anisometropic patients had less severe residual amblyopia at the end of part-time patching treatment. We found that the visual acuity of patients with FMNS improved with part-time patching but required longer treatment duration with poor stereoacuity at the end of the treatment. In the current study, we found although visual acuity improved in patients with and without FMNS within the first year of treatment, but the presence of FMNS was associated with a lower rate of visual acuity improvement. The treatment plateau occurs sooner in patients without nystagmus (average 39 weeks) compared to those with nystagmus without FMNS (60 weeks) and FMNS patients (> 2 years). We computed the treatment duration, which included the time when patching had to resume patching due to regression of the amblyopic eye visual acuity. We also had 4 (10%) children < 3 years of age. These could potentially result in greater treatment durations reported in our study than other studies^32–34^.

In our cohort, four patients did not have any visual acuity improvement after part-time patching. All these patients had mixed amblyopia with high anisometropia (> 4 diopters) and initiated patching treatment at age 5.5 years or older. These results agree with other studies that have reported high anisometropia and late age at therapy as risk factors for no improvement in visual acuity post patching treatment^26,34,42–47^.

### Effect of patching on stereoacuity

We analyzed the stereoacuity improvement in patients with and without FMNS and per the clinical type of amblyopia. We found that patients without nystagmus and anisometropic amblyopia had better stereoacuity at baseline, and both the visual acuity and stereoacuity improved with treatment. On the other hand, we found that patients with FMNS typically had no stereoacuity improvement despite the reduction of visual acuity deficit in the amblyopic eye with part-time patching treatment. This is in agreement with an observational study by Birch et al., where they reported that none of the amblyopic patients with normal stereoacuity had FMNS, whereas 67% of children with abnormal stereoacuity had FMNS. In contrast, all the children with nil stereoacuity had FMNS waveforms^26^. Also, in patients without FMNS, the stereoacuity improvement rate was slower, and the plateau time was higher than that of visual acuity improvement. This is likely due to the delayed development of fine stereoacuity, which is thought to still be immature at 5 years of age and with adult levels reaching between 6 and 9 years of age^48–51^.

### Regression

In our cohort, we found a regression risk of 17%. A few other studies have reported similar regression rates^17,18^. The PEDIG study 2004 has reported regression of 24% following patching therapy, with 6% of patients patching for more than 8 h/day^52^. Another PEDIG study reported regression of 7% within the first year of treatment cessation in older children between the ages of 7–12 years^53^. Other studies have found a regression rate of 24–27% in children after full-time occlusion therapy with a gradual taper^19^ versus an abrupt taper^54^. Studies have found that the risk of regression inversely correlates with the patient’s age at termination of treatment^19^. Other factors reported to be associated with regression are better visual acuity at the time of cessation of patching, greater visual acuity improvement during treatment, or previous regression^55,56^. In our cohort, one of our patients had experienced previous regression. All the patients except one who experienced regression were < 6 years of age. Overall we did not see a systematic trend between the risks of regression versus the level of visual acuity improvement. The differences between regression rates between our and other studies could be due to the varying ages of children in our cohort and the strictly part-time patching employed in our study. In our cohort, we found a significantly higher proportion of regression in patients with FMNS (50%) than in other groups (10%), with regression occurring only in those with strabismic/mixed form of amblyopia.

Increased risk of recurrence has been reported previously in patients with mixed amblyopia^15,17^. Nilsson et al. have reported the presence of microstrabismus alone as a risk factor of recurrence^18^. Holmes et al. and Rutstein and Fuhr found that excellent stereoacuity does not preclude the recurrence of amblyopia^55,57^. On the other hand, Bosworth and Birch reported the risk for persistent amblyopia was 2.2 times greater among children with nil stereoacuity^31^. Birch et al. have also described a higher rate of persistent amblyopia in patients affected by infantile esotropia (up to 60%) than accommodative esotropia^26^. In our cohort, we found that patients with strabismic or mixed amblyopia and FMNS both had greater chances to develop regression, whereas patients with strabismus without FMNS had similar levels of visual acuity and stereoacuity improvement as anisometropic amblyopes with a lower risk of regression.

Animal model studies have shown that disruption of binocularity during infancy is invariably associated with gaze instabilities, most often FMNS^6,58^. Tychsen and colleagues have shown in experiments that the prevalence and severity of FMNS increases with the longer duration of binocular decorrelation with 100% prevalence of FMNS in primates who are exposed to periods of binocular decorrelation that is equivalent to 3 months in humans. Tychsen has proposed that the binocular maldevelopment of the striate cortex is passed on to downstream extrastriate regions, namely the medial superior temporal area that drive conjugate gaze. The disruption results in a nasalward bias that is pathognomic of FMNS. Thus, animal model studies suggest that the development of FMNS is strongly associated with abnormal visual experience in infancy and can be used as a surrogate marker of the presence of amblyogenic risk factors/strabismus in the first year of life^59^.

Amblyopic patients both with and without nystagmus have fixation eye movement abnormalities compared to controls^23,37,60^. We have found that patients without nystagmus have a reduced frequency of physiologic microsaccades in the amblyopic eye compared to the fellow eye with increased inter-saccadic drifts in both the fellow and amblyopic eye. Patients with nystagmus with and without FMNS have increased slow phase velocities compared to inter-saccadic drift velocities in patients without nystagmus. We have also found that the slow phase velocities of FMNS patients are greater compared to patients with nystagmus without FMNS^60^. In the current paper, the analysis shows that FMNS is associated with a slower visual acuity rate of improvement, poor stereoacuity recovery, and higher regression rate with part-time patching treatment. We also found that patients with nystagmus but not FMNS tended to respond better to patching treatment than those with FMNS, even though eye movement abnormalities are still present. Thus, we speculate that patients with FMNS are likely to have early onset of amblyopia than those without FMNS, resulting in differences between the treatment outcomes in this cohort. The findings from our paper highlight the utility of fixational eye movement recordings in amblyopic patients in order to advance our understanding and field of knowledge of residual/recurrent amblyopia and to improve amblyopia therapy for specific types of amblyopia.

The study's main limitations are that the eye movement recordings were obtained at the end of treatment and that the treatment effect was determined based on a retrospective chart review. To reduce the impact of inaccurate data and individual testing biases, we excluded patients with incomplete data or that were not interpreted the same by at least two independent reviews. In our experience, these patients were noncompliant with treatment and did not follow up as frequently in our office per the recommendations. When evaluating the efficacy of patching treatment, it is essential to consider the effects of compliance. While we could not measure objective compliance, we did extract data obtained from clinical history to determine subjective compliance and included patients thought to be at least 50% compliant. Since the study is a longitudinal follow-up over a period of years, the visual acuity testing method differs depending on the techniques judged appropriate for the child's maturity. Regression was determined by two separate measurements with the same testing method to reduce the bias. The study reflects real-world scenario mimicking as encountered in clinical practice.

In summary, we examined the association between the presence of FMNS (confirmed on eye movement recordings) and the rate of improvement of visual acuity and stereoacuity and regression in amblyopia patients treated with part-time patching. We found that patients without nystagmus have a faster improvement of visual acuity and stereoacuity and plateaued sooner to reach their best possible visual acuity. FMNS is seen in patients with strabismic/mixed amblyopia, and the presence of FMNS was associated with a slower rate of improvement in visual acuity with poor/absent recovery of stereoacuity and a higher risk of regression. Thus, these results collectively highlight the link between the lack of binocular function and recurrent amblyopia. The current study's data suggest that eye movement characterization and quantification can play an essential role in amblyopia management. Children with FMNS and amblyopia should be observed closely with long-term follow-up and with a careful taper of the patching treatment. Future prospective studies, that measure FEMs at the time of treatment initiation will allow us to directly probe and understand the association of severity of visual acuity and stereoacuity deficits at the time of diagnosis and effects of different amblyopia treatments in patients with FMNS and other FEM abnormalities.

## Methods

### Study participants

Eye movement recordings were obtained in 80 amblyopic patients without any structural anomalies of the eye or neurologic disorders. The Cleveland Clinic Institutional Review Board approved the protocol and written informed consent was obtained from each participant or parent/legal guardian in accordance with the Declaration of Helsinki. The clinical parameters were extracted from a retrospective chart review for all the enrolled subjects. After review, we recruited 40 patients who had at least 12 months of follow up after initiating patching treatment and three sets of measurements, first at baseline, the second measurements between 3 and 6 months and third measurement between 9 and 12 months after initiating treatment, were included. Patients deemed to be at least 50% compliant were included in the study.

We categorized them based on the clinical type of amblyopia^61^ and on the fixational eye movements waveform characteristics (Table 1). Patients with manifest strabismus were treated according to the American Academy of Ophthalmology Preferred Practice Pattern^62^.

### Eye movement recording and analysis

A high-resolution video-based eye tracker (EyeLink 1000^®^, SR Research, Ontario, Canada) was used to measure binocular horizontal and vertical eye positions during binocular, fellow and amblyopic eye viewing conditions. All eye movement recordings were obtained at the end of patching treatment. An infrared permissive filter that blocked the visible light but allowed eye movement measurements of the non-viewing eye was used. Monocular calibration and validation were performed per the manufacturer’s guidelines. The subjects fixated their gaze on a circular target projected on the LCD screen on a white background (luminance 144 cd/m^2^) in a completely dark room for 45 s. The eye position data was analyzed after removal of blinks. The eye position signal was differentiated using MatlabTM (Mathworks, Natick, MA, USA) differential function and was further smoothened with the Savitzkey–Golay filter to measure eye velocity^22,35^. Fixational saccades and quick phases of nystagmus were identified using an unsupervised clustering method^35^. Drifts and slow phases were defined as epochs between fixational saccades and quick phases, respectively.

### Measurement of visual acuity, stereoacuity and strabismus

The clinical parameters were extracted from a retrospective chart review. The ages at the start of treatment and at follow up visits, visual acuity of the fellow and amblyopic eye and stereoacuity, cycloplegic refraction, strabismus angle measurements, and treatment compliance was noted. Visual acuity was measured in each eye monocularly, starting from the right eye, using the participant’s optimal spectacle correction with Snellen linear optotype. For patients younger than 6 years of age, per the child’s ability to perform the test, crowding bars HOTV optotypes were preferred and used over picture optotypes (Allen optotypes with crowding bars presented with commercially available computer-based system Accomodata Stimuli™). Visual acuity was measured at 20 feet distance, and the value was considered only if the patient could read all the letters (or symbols) of the line. Stereoacuity was measured with the Titmus Stereo Test at 40 cm. For analyses, visual acuity scores were converted into logMAR values, and stereoacuity scores in seconds of arc were converted to log arcsec values. For the purpose of analysis, subjects with no detectable (nil) stereoacuity were assigned a value of 7000″. There were only four patients that were diagnosed before their ability to perform any optotype and stereo-testing—they all had manifest strabismus with strong fixation preferencee (Table 1, patients n. 1, 25, 33, 38).

These four patients were all assigned as having severe amblyopia with absent stereoacuity. The strabismus was assessed in the primary position at distance and near measured by alternate and simultaneous prism cover tests and Hirschberg and Krimsky tests in younger patients. The clinical categorization of amblyopia subtype and severity at the time of diagnosis was based on PEDIG studies^32,33,47,63^.

### Amblyopia treatment and measurement

The treatment comprised of part-time occlusion (2–6 h/day), prescribed per the severity of amblyopia^32,64^. Strabismic patients were diagnosed before other groups (anisometropic vs strabismic vs mixed: 71.7 ± 15.9 vs 23.7 ± 13.4 vs 59.7 ± 15.1 months, Kruskal–Wallis Test p = 0.016), while no differences in presentation time to start of patching treatment were observed grouped per fixational eye movement characteristics (no nystagmus vs nystagmus no FMNS vs FMNS: 68.2 ± 21.1 vs 62 ± 23.9 vs 49.8 ± 28.1 months, Kruskal–Wallis Test p = 0.346). Investigators judged patching compliance to be good (> 50%), fair (26–50%), or poor (≤ 25%), based on discussions with the parents documented in the chart comparing the number of hours prescribed and the ones declared by the parents including the number of daily and weekly hours of patching treatment^65^.

Visual acuity and stereoacuity from the start to the end of treatment were computed as a function of the clinical type and fixation eye movements characteristics. We also calculate the rate of visual acuity and stereoacuity change within the first year. The rate of improvement was analyzed as a function of the clinical type of amblyopia and fixational eye movements characteristics. Patients who did not have any improvement in visual acuity on two consecutive visits were not included in this analysis as they were considered to be non-responsive to patching treatment. These patients opted for either atropine penalization or stopped the treatment. The patching treatment was continued beyond the first year per the clinical management. Patching was discontinued if the visual acuity had stabilized with no further improvement or deterioration ≥ 2 consecutive visits ≥ 6 weeks apart in patients with at least 50% compliance. Patients who were patching 6 h/day were gradually weaned of treatment as the visual acuity improved^52^. After the patching treatment was discontinued, the visual acuity and stereoacuity measurements obtained at 3 months interval were recorded to detect regression. Regression was defined as a drop in visual acuity by 2 lines as obtained by two separate measurements (on the same or different day) from the previous visit, and treatment was restarted in these patients^52^. The duration of patching treatment (treatment plateau in months) required to reach the best possible visual acuity and stereoacuity with no further improvement or regression was analyzed as a function of clinical subtype and fixational eye movements characteristics.

### Statistical analysis

All analyses were performed in SPSS and GraphPad Prism 7 (La Jolla, CA, USA). A t test, Kruskal–Wallis Test, Mann Whitney U Test and one-way ANOVA were used to compare the demographics and baseline characteristics amongst the groups.

Mixed-effects regression models with random intercepts to test the hypothesis of a difference in the rate of change in the visual acuity and stereoacuity between patients per fixational eye movements characteristics was used. The hypothesis was assessed by comparing the slope of change in the LogMAR visual acuity over the 12-month period per the fixational eye movement characteristics with a negative slope reflecting visual acuity improvement. The slope of change in the log arc seconds stereoacuity over the 12-month period per the fixational eye movement characteristics was compared with a negative slope reflecting stereoacuity improvement. We also performed linear regression separately for each patient (visual function versus treatment duration) and extracted the beta coefficient (degree of visual function change for every 3 months of patching treatment). We also report the average beta coefficient values for a given subgroup. A similar analysis per the different clinical types of amblyopia: anisometropic and strabismic/mixed was done (patients with strabismus and mixed amblyopia were pooled together as there were few strabismic patients n = 3). One-way ANOVA was used to compare the total improvement of visual acuity and stereoacuity and treatment duration grouped per the fixational eye movement characteristics. A t test was used to analyze these parameters as a function of the clinical type of amblyopia (strabismic/mixed versus anisometropic patients).
